# Development and validation of a nomogram model for individualized prediction of hypertension risk in patients with type 2 diabetes mellitus

**DOI:** 10.1038/s41598-023-28059-4

**Published:** 2023-01-23

**Authors:** Jing Yang, Xuan Wang, Sheng Jiang

**Affiliations:** Department of Endocrinology, The First Affiliated Hospital of Xinjiang Medical University, Urumqi, 830017 China

**Keywords:** Endocrinology, Risk factors

## Abstract

Type 2 diabetes mellitus (T2DM) with hypertension (DH) is the most common diabetic comorbidity. Patients with DH have significantly higher rates of cardiovascular disease morbidity and mortality. The objective of this study was to develop and validate a nomogram model for the prediction of an individual's risk of developing DH. A total of 706 T2DM patients who met the criteria were selected and divided into a training set (n = 521) and a validation set (n = 185) according to the discharge time of patients. By using multivariate logistic regression analysis and stepwise regression, the DH nomogram prediction model was created. Calibration curves were used to evaluate the model's accuracy, while decision curve analysis (DCA) and receiver operating characteristic (ROC) curves were used to evaluate the model's clinical applicability and discriminatory power. Age, body mass index (BMI), diabetic nephropathy (DN), and diabetic retinopathy (DR) were all independent risk factors for DH (P < 0.05). Based on independent risk factors identified by multivariate logistic regression, the nomogram model was created. The model produces accurate predictions. If the total nomogram score is greater than 120, there is a 90% or higher chance of developing DH. In the training and validation sets, the model's ROC curves are 0.762 (95% CI 0.720–0.803) and 0.700 (95% CI 0.623–0.777), respectively. The calibration curve demonstrates that there is good agreement between the model’s predictions and the actual outcomes. The decision curve analysis findings demonstrated that the nomogram model was clinically helpful throughout a broad threshold probability range. The DH risk prediction nomogram model constructed in this study can help clinicians identify individuals at high risk for DH at an early stage, which is a guideline for personalized prevention and treatments.

## Introduction

Diabetes is a metabolic disease with a high global prevalence. About 42.2 million adults worldwide have diabetes, which is projected to reach 642 million by 2045, according to the World Health Organization^1^. Data from the Chinese National Diabetes and Metabolic Disorders Study^2^, the Chinese Chronic Non-Communicable Disease Surveillance System^3^, and the Chinese Survey of Chronic Diseases and Their Risk Factors^4^with large population samples showed that the prevalence of diabetes in China was 9.7%, 11.6%, and 10.9%, respectively. Type 2 diabetes mellitus (T2DM) often coexists with hypertension (DH). In a cross-sectional study conducted in China from 2010 to 2012, 25,817 type 2 diabetic patients were included. The results showed that 59.8% of diabetic patients combined with hypertension^5^. A retrospective study based on the Chinese population in Taiwan found that the proportion of hypertension in T2DM patients was also very high (53.9% of insulin users, and 61.3% of non-insulin users)^6^.

Diabetes combined with hypertension is associated with a higher risk of macrovascular and microvascular disease. A 10.9-year follow-up study in Shanghai found that the coexistence of diabetes and hypertension increased cardiovascular disease risk up to 3.34 times^7^. Furthermore, T2DM patients with hypertension are at higher risk of developing diabetic complications such as retinopathy and diabetic nephropathy, which increases the burden on families and society^8,9^. T2DM combined with hypertension has a much higher mortality rate than T2DM patients without hypertension so early treatment and active prevention of cardiovascular diseases have become a hot research topic in recent years^10^.

The exploration of independent risk factors for type 2 diabetes mellitus complicated by hypertension and the taking of effective preventive measures could effectively reduce the risk of diabetes^11^. As far as I know, few studies have been done on predictive models and risk factor analyses of hypertension in type 2 diabetes. Xue et al. found age and obesity to be determinants in a study of risk factors for hypertension in patients with type 2 diabetes in a Chinese community, which is similar to our findings, but unfortunately, they did not include diabetes-related complications in the model, which is formally an innovation of our model^12^.

The purpose of this study was to analyze risk factors for type 2 diabetes associated with hypertension using available clinical data. In order to identify T2DM patients with the possibility of hypertension, a nomogram model was constructed based on independent risk factors. Thus, it can effectively prevent hypertension; reduce the incidence of cardiovascular events in diabetic patients, and improve the quality of life of patients.

## Methods

### Study design and participants

**Figure 1:**
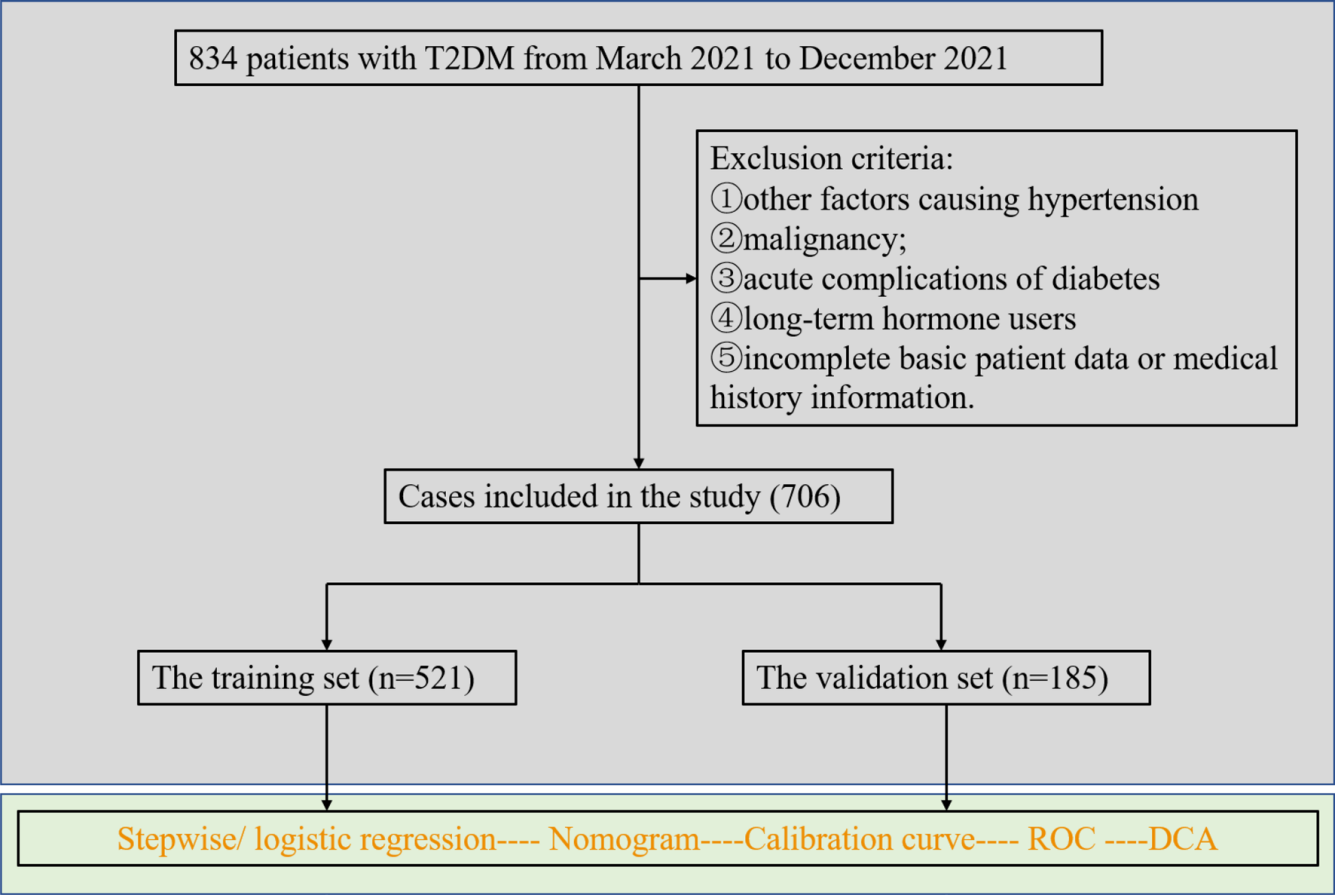
Flowchart of study participants.

We reviewed data from 706 T2DM patients hospitalized in the Endocrinology Department of First Affiliated Hospital of Xinjiang Medical University from March 2021 to December 2021. All 706 patients with type 2 diabetes were diagnosed according to the relevant diagnostic criteria in the “Guidelines for the Prevention and Control of Type 2 Diabetes in China (2019)”^13^. Hypertension was diagnosed according to the diagnostic criteria of the Chinese Guidelines for the Management of Hypertension 2019^14^: systolic blood pressure ≥ 140 mmHg and/or diastolic blood pressure ≥ 90 mmHg measured three times on non-same days (usually 2 weeks apart) without any anti-hypertensive medication. Exclusion criteria: ①other factors causing hypertension (renal disease, pheochromocytoma, primary aldosteronism); ②malignancy; ③acute complications of diabetes (diabetic ketoacidosis, hyperglycemic hyperosmolar coma); ④long-term hormone users; ⑤incomplete basic patient data or medical history information (Fig. 1). The study protocol complied with the principles of the Declaration of Helsinki. The Ethics Committee of the First Affiliated Hospital of Xinjiang Medical University approved the study and agreed to waive the written informed consent, because the study was retrospective and all the data was collected in an electronic medical record system. All methods were performed in accordance with the relevant guidelines and regulations.

### Data collection

We collected clinical information about patients from the electronic case system including age, gender, duration of diabetes, body mass index (BMI), and presence of chronic diabetes complications (diabetic peripheral neuropathy, diabetic retinopathy, diabetic nephropathy). In addition, we also collected biochemical indicators including fasting blood glucose (FBG), glycated hemoglobin (HbA1c), serum creatinine (Scr), triacylglycerol (TG), and high-density lipoprotein cholesterol (HDL-C). Biochemical parameters were measured by a Beckman Coulter automatic biochemical analyzer (Beckman Coulter, Inc, USA). Measurement of Scr concentration was performed by the Jaffé method; HDL-C was measured by the direct method; TG was measured using the GPO-POD method, and FBG was measured with the hexokinase method. The estimated glomerular filtration rate (eGFR) was calculated using the simplified creatinine-based Modification of Diet in Renal Disease formula^15,16^.

The diagnosis of DN is determined according to the ratio of microalbumin to creatinine (ACR). In this study, microalbuminuria (30 ≤ ACR ≤ 300 mg/g) and clinical albuminuria (ACR > 300 mg/g) are both represented as DN^17^. We used Optos Daytona ultra-wide field fundus camera (scanning laser ophthalmoscope, Optos, Daytona, UK) to image all subjects and evaluate retinopathy. If fundus photography shows that the subjects have soft exudates, hard exudates, hard exudates, retinal hemorrhage points, neovascularization, or vitreous hemorrhage, it is defined as DR^18^. The diagnosis of DPN meets the diagnostic criteria for DPN in the Chinese Guidelines for the Prevention and Treatment of Type 2 Diabetes Mellitus (2019 edition)^13^.

### Statistical analysis

Statistical analysis was performed using R software (version 4.1.1; https://www.r-project.org). Univariate logistic regression analysis was used to analyze clinical variables, and variables included in the multivariable logistic regression model were determined by univariate logit regression results and professional knowledge. The statistically significant indicators were filtered by stepwise regression analysis. The stepwise regression with the minimum Akaike information criterion was used to select variables for inclusion in the nomogram^13^. In addition, the Akaike information criterion is a standard for measuring the goodness of fit of statistical models, which can measure the complexity of the estimated model and the goodness-of-fit of the model fitted^19^. Once risk factors were screened, the rms package^20^ in R software was used to establish the risk nomogram model for DH.

The bootstrap method was used to internally verify the nomogram model 1000 times, and the concordance index (C-index) was computed to assess the discrimination of the nomogram model. C-index0.50–0.70 was the low precision, 0.71–0.90 was the average quasi-accuracy^21^, and above 0.90 was the high precision. Consistency was tested by plotting the calibration curves between the predicted and actual results. The model discrimination was evaluated by plotting receiver operating characteristic (ROC) curves with the pROC package. ROC curve is a monotonically increasing curve constructed by connecting the true positive rate and false positive rate values at different cut points or thresholds^22^. Area AUC values under the curve can be used as indices to measure the diagnostic effect. In general, the larger the area value, the better the classification approach will be. Receiver operating characteristic curves are widely used in medical diagnostics^23,24^. The only concern of the ROC curve, however, is the accuracy of the predictive model, which does not help to determine the usefulness of the real clinical model.

Clinical decision curve analysis (DCA) is a simple and easy-to-understand mathematical model to assess the usability and effectiveness of predictive models. One of the basic principles of DCA generation is that the relative loss values of false positives and false negatives can be expressed as threshold probability. Many papers^25–27^ have adopted, introduced, and recommended the use of DCA in recent years, which can better assess the clinical benefit of the ROC curve-based model. Therefore, we used clinical decision curve analysis to assess the clinical efficacy of this model. As further validation of the stability of this prediction model, we internally validated the whole data set and obtained the C-index and area under the curve (AUC), and plotted the DCA as well.

### Ethics approval and consent to participate

The study was conducted by the Declaration of Helsinki. The Ethics Committee of the First Affiliated Hospital of Xinjiang Medical University approved the study. Because it was a retrospective study, written informed consent was waived.

## Results

### Patient characteristics

A total of 706 subjects were included in the study, of whom 402 (56. 94%) were hypertensive patients and 304 (43. 06%) were non-hypertensive patients. There were 438 males (62.04%) and 268 females (37.96%). We divided the study population into a training set (n = 521) and a validation set (n = 185). Differences in age, FBG, IGF-1, and DN between the 2 groups were statistically significant (P < 0.05). There was no statistically significant difference between the two groups in comparing other indicators (P > 0.05) (Table 1).

**Table 1:** Baseline characteristics of training and validation sets.

Characteristic	Training set (n = 521)	Validation set (n = 185)	*P*-value
Age [years]	56.84 ± 12.38	54.56 ± 11.13	0.021
Gender (n, %)			
Male	317 (60.8)	121 (65.4)	0.313
Female	204 (39.2)	64 (35.6)	
Duration of diabetes [years]	8.76 ± 7.29	8.81 ± 7.30	0.945
BMI [kg/m^2^]	26.02 ± 4.62	26.06 ± 3.54	0.909
Hypertension (n, %)			
Yes	300 (57.6)	102 (55.1)	0.624
No	221 (42.4)	83 (44.9)	
Diabetes complications			
DN (n, %)			
Yes	133 (34.3)	62 (33.5)	0.046
No	388 (65.7)	123 (66.5)	
DPN (n, %)			
Yes	298 (57.2)	116 (62.7)	0.223
No	223 (42.8)	69 (37.3)	
DR (n, %)			
Yes	313 (60.1)	125 (67.6)	0.778
No	208 (39.9)	60 (22.4)	
Lifestyle habits			
Smoking (n, %)			
Never	354 (67.9)	114 (61.6)	0.141
Ever/current	167 (32.1)	71 (38.4)	
Consuming alcohol (n, %)			
Never	364 (69.9)	122 (65.9)	0.37
Ever/current	157 (30.1)	63 (34.1)	
Biochemical tests result in characteristics			
FBG [mmol/L]	7.93 ± 2.67	7.15 ± 2.13	< 0.001
2H-OGTT [mmol/L]	16.69 ± 4.48	16.55 ± 11.06	0.868
HbA1c [%]	8.75 ± 2.21	8.71 ± 1.88	0.833
eGFR [ml/min/1.73 m^2^]	94.13 ± 21.08	96.08 ± 22.26	0.300
Scr [μ mol/L]	71.74 ± 24.94	70.28 ± 23.97	0.491
TG [mmol/L]	2.15 ± 1.97	2.10 ± 2.03	0.777
HDL-C [mmol/L]	0.98 ± 0.27	0.99 ± 0.35	0.489

### Feature selection

Univariate logistic regression analysis was performed to analyze clinical variables in patients with DH. The relevant influencing factors of DH included age, BMI, duration of diabetes, DPN, DR, DN, IGFBP-3, eGFR, and Scr (Table 2).

**Table 2:** Predictors identified by univariate logistic regression analysis.

Variables	Univariate logistic regression		
	*β*	OR (95% CI)	*P*-value
Age [years]	0.071	1.073 (1.055–1.093)	< 0.001
BMI [kg/m^2^]	0.075	1.078 (1.031–1.129)	0.001
Diabetes duration [years]	0.042	1.042 (1.017–1.069)	0.001
DPN [yes vs. no]	0.559	1.750 (1.231–2.493)	0.002
DR [yes vs. no]	0.814	2.258 (1.580–3.239)	< 0.001
DN [yes vs. no]	1.368	3.926 (2.493–6.354)	< 0.001
IGFBP-3 [μ g/mL]	− 0.151	0.859 (0.743–0.992)	0.039
eGFR [ml/min/1.73 m^2^]	− 0.034	0.967 (0.956–0.977)	< 0.001
Scr [μ mol/L]	0.015	1.015 (1.006–1.024)	0.001

### Construction of the nomogram prediction model

**Figure 2:**
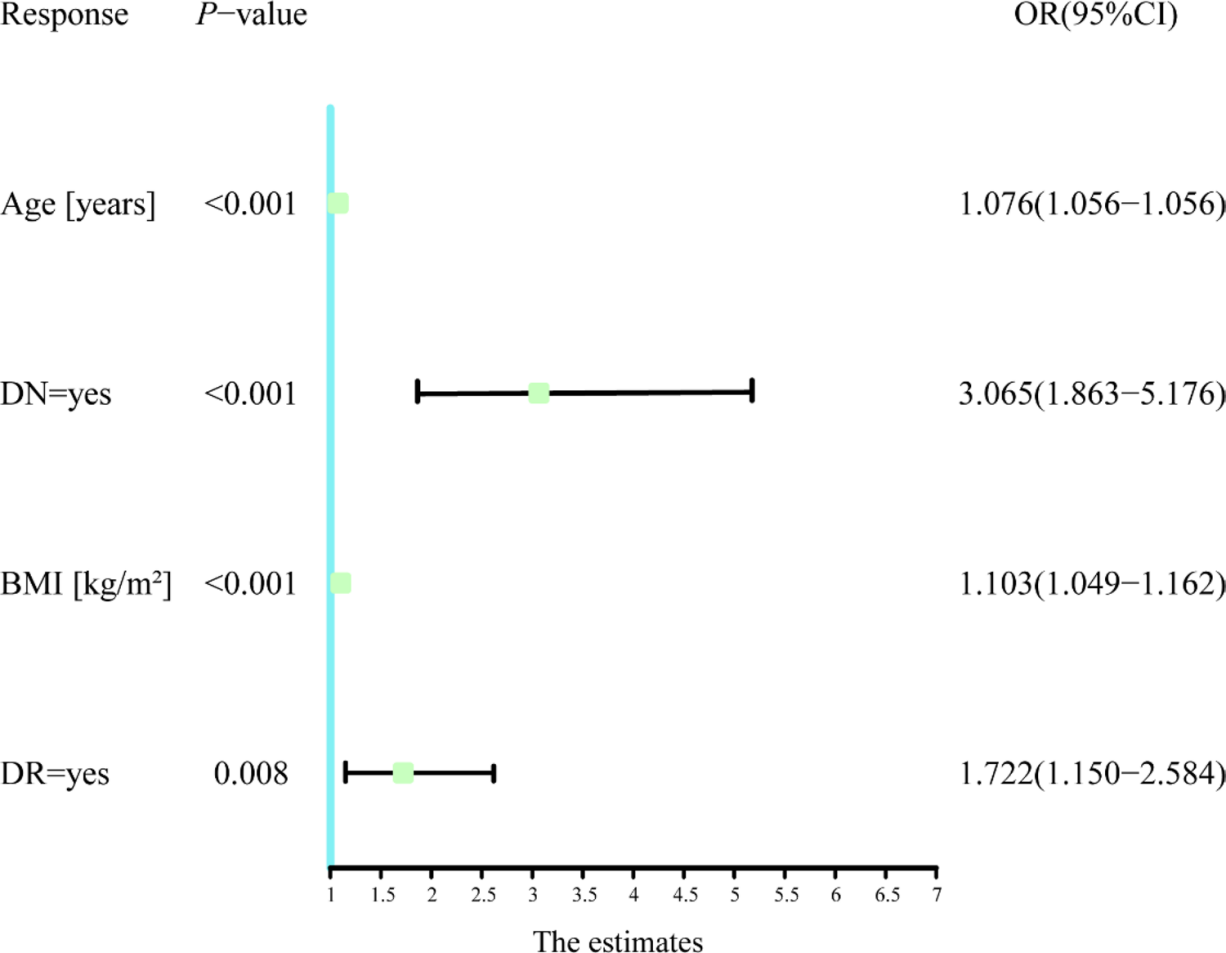
Forest plot for building the OR of the 4 variables of the model.

**Figure 3:**
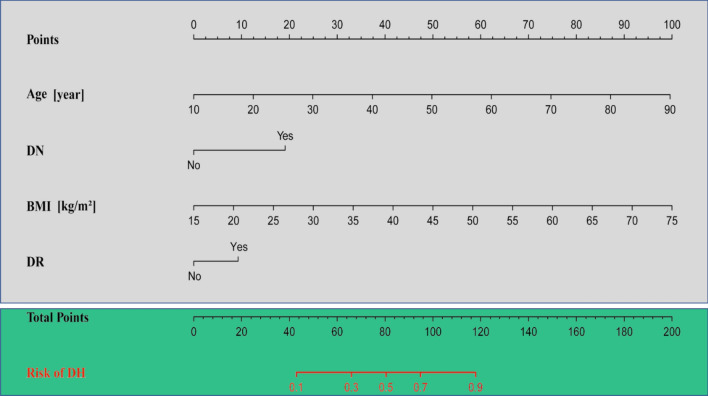
Nomogram for predicting DH. Age, DN, BMI, and DR are the variables of the prediction model.

Multivariate logistic regression analysis was used to analyze variables that were significant in the univariate logistic analysis. The results showed that age, BMI, DR, and DN were independent risk factors for DH (Table 3). We visually present the results of multivariate logistic regression using forest plots (Fig. 2). The nomogram model was established using predictive variables filtered by multivariate logistic regression (Fig. 3). Using the rule above the nomogram corresponding to each risk factor, a single score of that factor could be obtained. Every single score was summed, and the total score corresponded downward to the probability of DH in this patient. Scores increased by 1.25 points for each 1-year increase in age, 20 points if DN was present, 1.67 points for every 1 kg/m^2^ BMI increase, and 10 points for DR.

**Table 3:** Multivariate logistic regression analysis of step AIC selection to construct a nomogram model.

Variables	Multivariate logistic analysis		
	*β*	OR (95% CI)	*P*-value
Age [years]	0.730	1.076 (1.056–1.097)	< 0.001
DN = yes	1.120	3.065 (1.863–5.176)	< 0.001
BMI [kg/m^2^]	0.098	1.103 (1.049–1.162)	< 0.001
DR = yes	0.544	1.722 (1.150–2.584)	0.008

The points on the nomogram represent the score of each variable, and the total points represent the total score of all variables after summing the scores. The red line represents the probability of DH occurrence, and the vertical line extending from total points down to the red line is the probability of DH occurrence.

**Figure 4:**
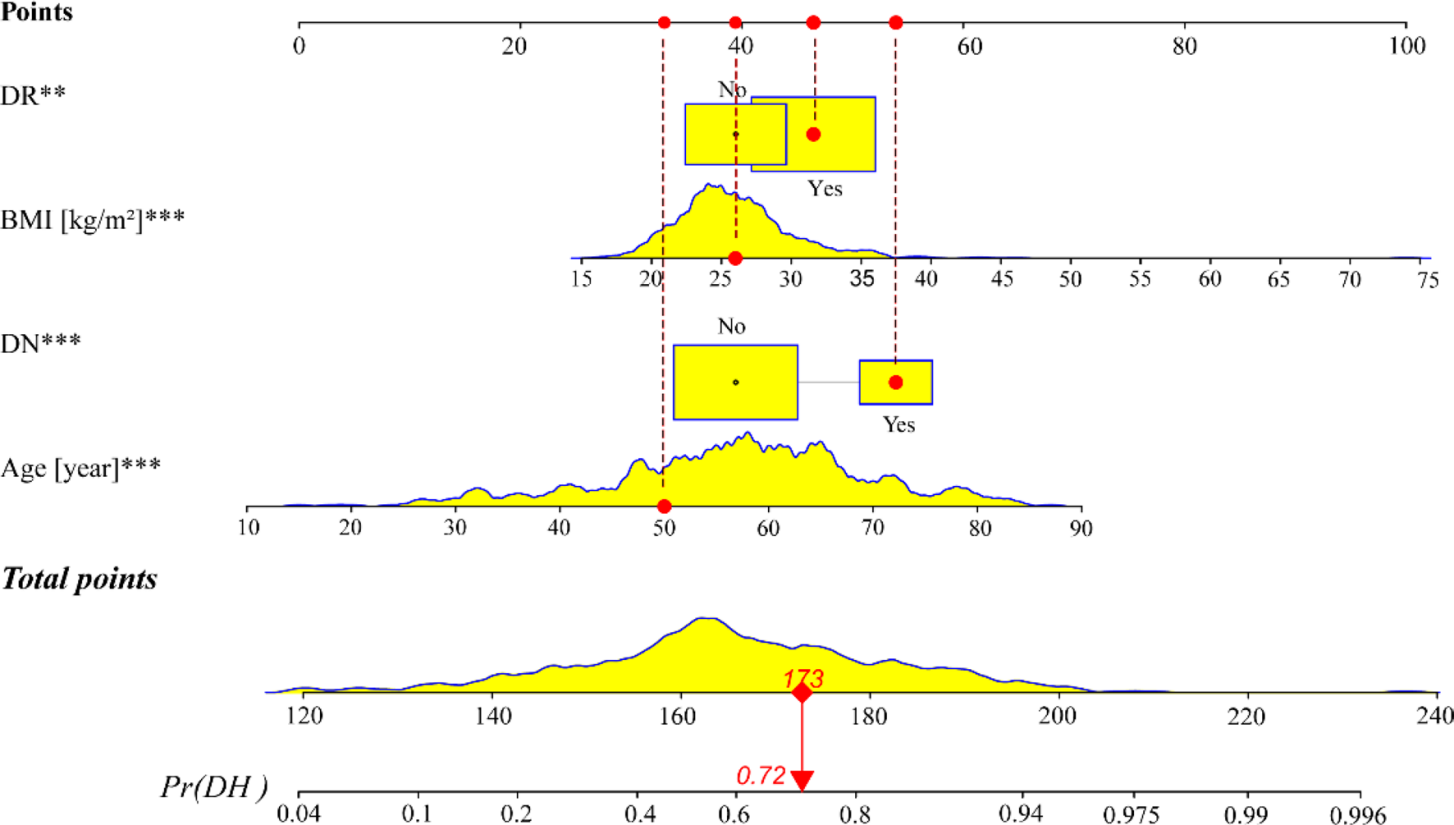
An example of a nomogram for DH. The yellow areas in the graph represent the distribution of the variables in the cohort. DN and DR are the highescoringsing, with relatively low scores for BMI and age.

Take an example of nomogram usage, the red in the figure represents the risk of hypertension in the first type 2 diabetes patient among 706 patients in the present study: age (50 years old), BMI (26 kg/m^2^), DN (yes), RD (yes). When the four index scores are summed, the total patient score is 173. A vertical line is drawn at the coordinate of the total points 173, and the pretest probability of the corresponding is approximately 72%. In other words, this patient has a 72% risk of hypertension (Fig. 4), and it is recommended that in clinical practice, high priority be given to the prevention of hypertension in this group of patients.

### Performance of the DN risk nomogram in the cohort

**Figure 5:**
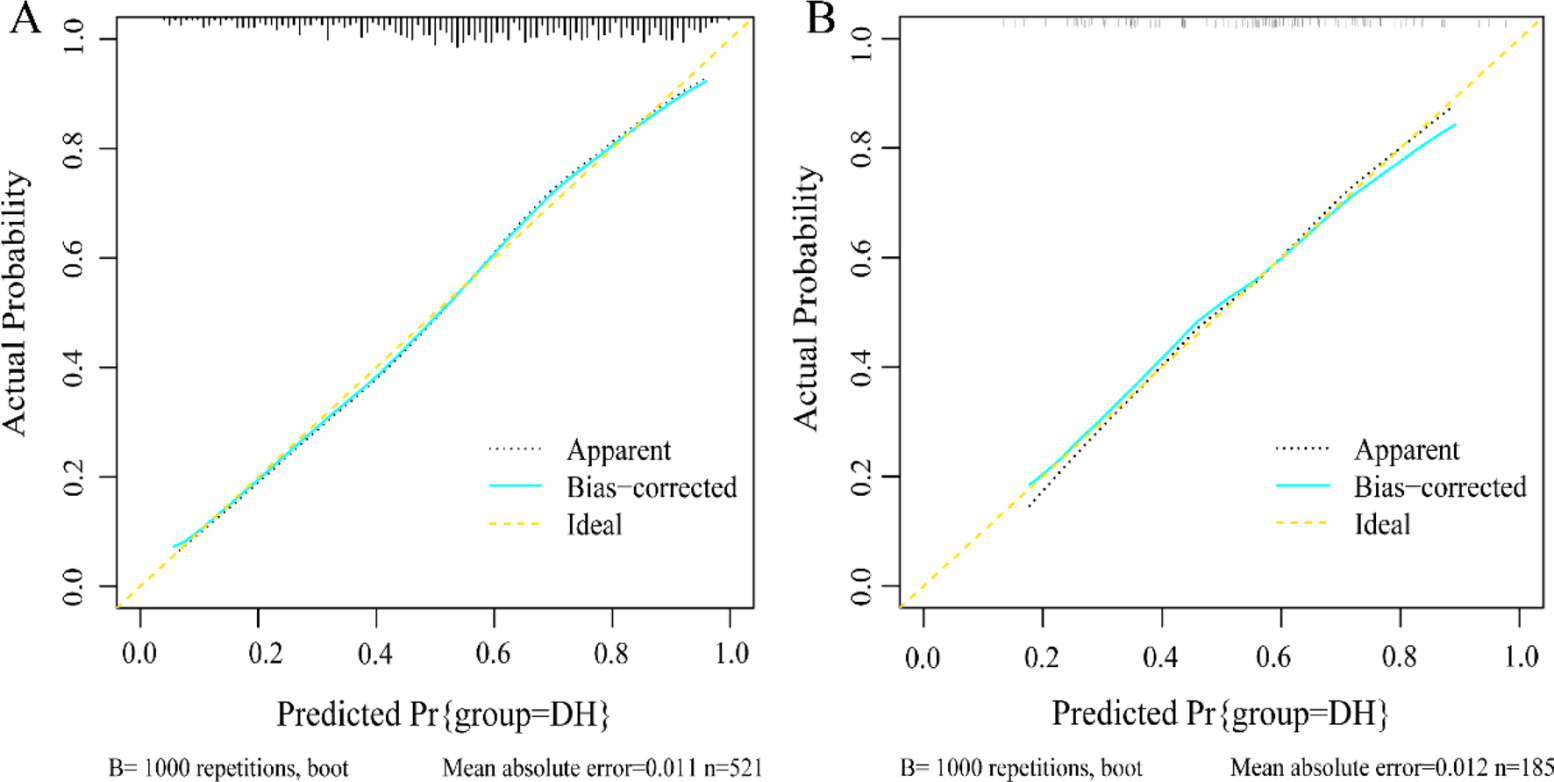
Calibration curves of the nomogram. (**A**) represents the calibration curve of the training set, and (**B**) represents the calibration curve of the validation set. The x-axis represents the predicted probability of DH. The y-axis represents the actual diagnosed DH. the yellow dashed line represents the perfect prediction with the same predicted probability as the actual probability. The black dashed line represents the performance of the nomogram and the green solid line represents the performance of the model after calibration. The closer the calibration curve of the model is to the yellow dashed line, the better the model prediction is represented.

**Figure 6:**
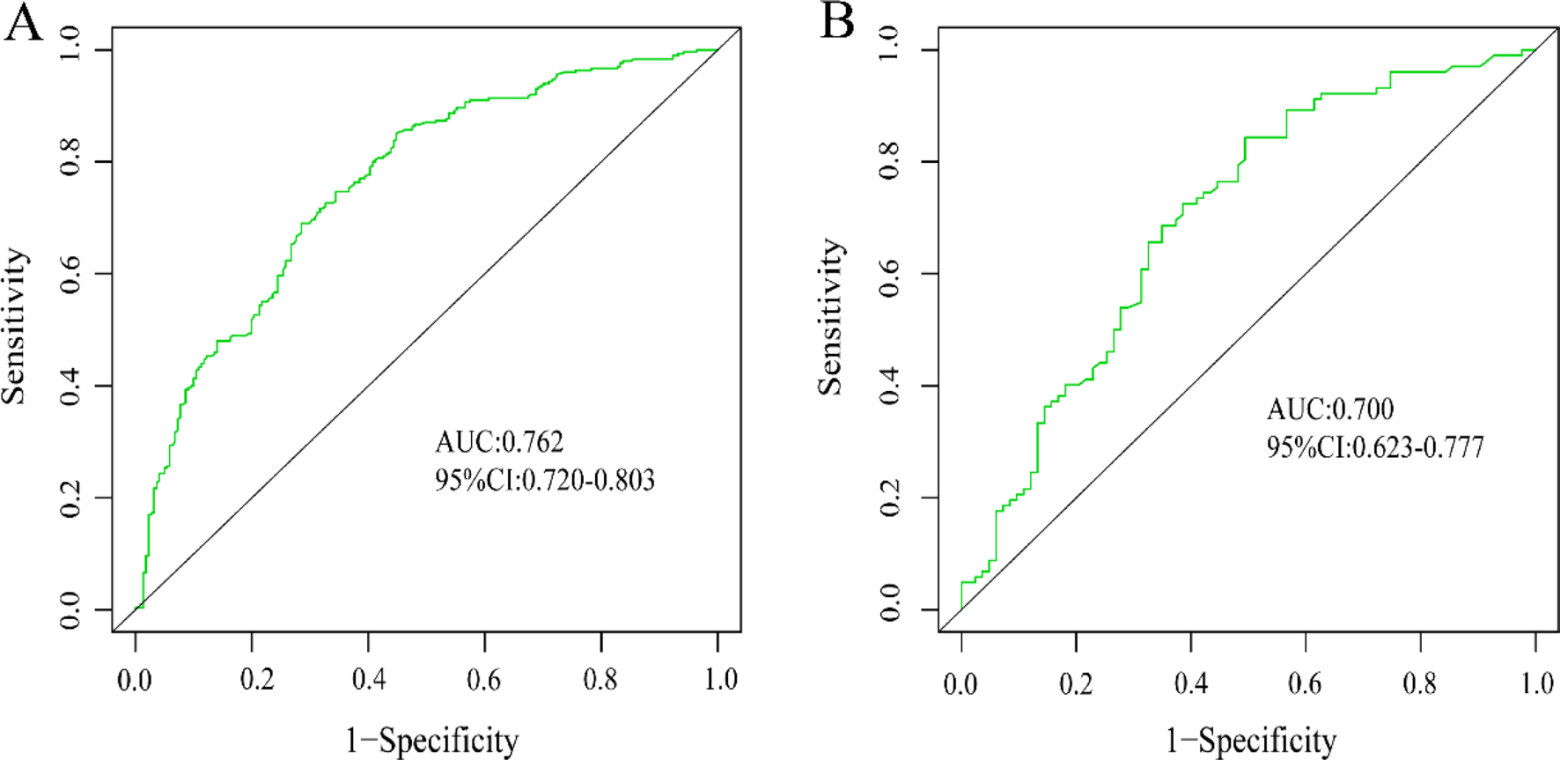
The ROC curves of the nomogram model. (**A**) indicates the ROC curve of the training set, and (**B**) indicates the ROC curve of the validation set. The x-axis represents 1-specificity, and the y-axis represents sensitivity. The part below the green line is the area under the ROC curve of the model.

**Figure 7:**
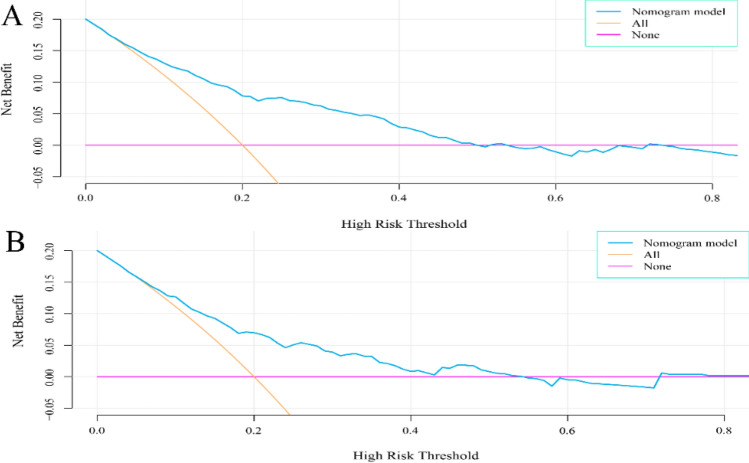
Clinical decision curves for the nomogram model. (**A**) indicates the decision curve for the training set, and (**B**) indicates the decision curve for the validation set. the x-axis shows the threshold probability, and the y-axis shows the net benefit. The purple line represents that all patients did not experience DH, and the orange line indicates that all patients experienced DH. the blue line represents the model; the decision curves show that the model is clinically beneficial within a relatively large range of threshold probabilities.

In this study, the C-index of the training set and validation set were 0.762 and 0.700 respectively, which indicated that the prediction of this nomogram was in good accordance with the real observation. For predicting the risk of internal hypertension Boostrcross-validationion, the nomogram was run 1000 times, and the mean error rates of the two models were 0.011 (Fig. 5A) and 0.012 (Fig. 5B) respectively, indicating that the nomograms had superior stability. The AUC of the training set is 0. 762 (95% CI 0.720–0.803). The AUC of the DH nomogram prediction model for the validation set was 0.700 (95% CI 0.623–0.777). In addition, the AUC of both the training set (Fig. 6A) and the validation set (Fig. 6B) were between 0.7–0.9, which indicates that the pretested models had good discrimination in both the training set and the validation sets. Clinical DCA suggests that the nomogram model is beneficial whether in the training set (Fig. 7A) or the validation set (Fig. 7B) within a wide threshold probability range.

## Discussion

The prevalence of hypertension in patients with type 2 diabetes was 56.9% which was 1.32 times higher than that in patients without hypertension in this study. This finding is similar to the findings of hypertension prevalence in type 2 diabetic patients from a universal health checkup conducted in Urumqi, Xinjiang, China in 2018^12^. Therefore, to better predict the risk of combined hypertension in patients with type 2 diabetes, we constructed a hypertension risk nomogram prediction modeAThe nomogram is a visual tool for predicting the probability of occurrence of events based on statistical models with multiple factors^28^. Over the past few years, the use of nomograms to construct predictive models has become widely adopted^29^. The ability of clinical nomogram prediction models to more accurately predict individual disease risk based on different patient conditions may aid clinical decision-making. Stepwise regression results combined with multifactorial logistic regression model analysis in this study showed that age, BMI, DN, and RD were independent risk factors for DH.

The results of this study showed that age was a risk factor for hypertension. Hypertension prevalence increased with age^30,31^. This can be mainly due to the gradual hardening of the large arteries as we age and the gradual loss of vascular elasticity, leading to increased blood pressure^32^. Variability in blood pressure also increases with age, and in addition to these age-related factors, comorbidities such as dyslipidemia and elevated blood glucose increase with aging. Over time, vascular endothelial cell dysfunction occurs, with a decline in the renin–angiotensin–aldosterone system and aging of the kidney, which leads to restricted arterial dilation^33^. Therefore, patients with type 2 diabetes need to have their blood pressure monitored more closely as they age, and if hypertension is detected, it needs to be controlled with appropriate medications based on lifestyle interventions to reduce the risk of heart failure, stroke, myocardial infarction, and death.

In the prediction model, BMI was an influential factor in the development of hypertension, which is consistent with previous studies^34^. In addition, Huang et al. also found that BMI was an independent risk factor for hypertension risk^35^. Similarly, a study conducted in Taiwan found that BMI is the main and independent determinant of blood pressure and hypertension in adult type 2 diabetes patients in Taiwan^36^. BMI is currently used clinically as an indicator of overweight or obesity. Compared to other commonly used measures of obesity, BMI is the most sensitive body indicator to predict high blood pressure in adults^37^. At the pathophysiological level, the core mechanism of obesity associated with T2DM is insulin resistance. Similarly, the mechanisms associated with the development of obesity and hypertension include insulin resistance, and the remaining mechanisms include inflammation, oxidation, active stress, and excessive activation of the sympathetic nervous system and the renin–angiotensin–aldosterone system^38–40^. These factors interact to cause systemic hemodynamic alterations. The obese population is at high risk for hypertension and obesity promotes atherosclerosis^41^. Pathophysiological changes associated with obesity include increased arterial stiffness^42^, as well as altered reflex sensitivity to pressure receptors, which is also responsible for increased variability in blood pressure. Therefore, patients with type 2 diabetes need good lifestyle interventions^43^, such as smoking cessation and alcohol restriction, moderate exercise, low-salt and low-fat and multi-vitamin diets, which can effectively maintain a healthy body state and reduce weight. These measures can effectively improve insulin resistance, which not only facilitates blood glucose control but also has beneficial effects on blood pressure and blood lipids^44^.

Microvascular complications are the leading cause of mortality in diabetic patients, and DN and DR are the most severe microvascular complications. Common pathogenesis shared by DR and isare high glucose toxicity, free radicals, oxidative stress of inflammatory mediators, and abnormal polyols metabolism, all of which play very important roles^45^. Most people with type 2 diabetes will develop DN and DR if they have had diabetes for more than 20 years. As chronic hyperglycemia impairs vascular endothelial cell function, vascular inflammation occurs, resulting in decreased peripheral vascular elasticity^46^ and increased peripheral resistance. The kidney is both an important organ for blood pressure regulation and a major target organ for hypertension damage. DN causes disorders in salt and water regulation in the kidney, especially abnormalities in the renin-angiotensin system^47^ and abnormalities in the sympathetic nervous system^48^. All of the above factors interact with each other, resulting in the development and progression of hypertension^49^, whereas the presence of hypertension may further worsen DR and DN^50^.

There are some limitations in this study: firstly, the data in this study were obtained from a single center and were retrospective, which may have some information bias and patient selection bias; a prospective study design is necessary for the future. Secondly, in terms of model validation, only internal validation was performed, and further validation of the model with a large multicenter sample is needed to assess the applicability of the model in the future. Finally, we did not collect information about coronary heart disease, stroke, lower limb amputation, heart failure, and other diseases highly related to hypertension, which may affect the prediction ability of our model.

## Conclusion

In conclusion, this study constructed a risk prediction model with a good discriminatory ability for DH, including the 4 risk factors of age, BMI, DN, and DR. The model had good consistency, discrimination, and clinical applicability, and the risk of hypertension was rapidly calculated by the Nomogram model, which can guide the clinical formulation of targeted treatment and interventions.

## Data Availability

The data and R code used in this current study are available from the corresponding authors at a reasonable request.

## Abbreviations

T2DM: Type 2 diabetes mellitus
DH: Type 2 diabetes mellitus with hypertension
DCA: Decision curve analysis
ROC: Receiver operating characteristic
BMI: Body mass index
DN: Diabetic nephropathy
DR: Diabetic retinopathy
DPN: Diabetic peripheral neuropathy
FBG: Fasting blood glucose
HbA1cl: Glycated hemoglobin
Scr: Serum creatinine
TG: Triacylglycerol
HDL-C: High-density lipoprotein cholesterol
eGFR: Estimated glomerular filtration rate
IGFBP-3: Insulin-like growth factor binding protein-3
IGF-1: Insulin-like growth factor-1
C-index: Concordance index
AUC: Area under the curve
